# Association of postural orthostatic tachycardia syndrome, hypermobility spectrum disorders, and mast cell activation syndrome in young patients; prevalence, overlap and response to therapy depends on the definition

**DOI:** 10.3389/fneur.2025.1513199

**Published:** 2025-04-25

**Authors:** Lilian Yao, Kavya Subramaniam, Katherine M. Raja, Abi Arunachalam, Aubrey Tran, Tripti Pandey, Sahana Ravishankar, Sahan Suggala, Caitlyn Hendrickson, Andrew J. Maxwell

**Affiliations:** Heart of the Valley Pediatric Cardiology, Pleasanton, CA, United States

**Keywords:** dysautonomia, POTS, mast cell activation syndrome, Ehlers-Danlos syndrome, hypermobility spectrum disorder, autonomic neuro-immune axis dysfunction

## Abstract

**Background:**

The close association of syndromes of orthostatic intolerance with and without postural orthostatic tachycardia syndrome (POTS) with Joint Hypermobility Disorders (JHD) including Hypermobility Spectrum Disorder (HSD) and hypermobile Ehlers Danlos Syndrome (hEDS) and with Mast Cell Activation Syndrome (MCAS) is now firmly established. However, the prevalence of each entity relative to the other is not well established and is affected greatly by the various definitions used for each syndrome. Use of restricting definitions for each syndrome can be problematic in the clinical setting as it under-estimates the presence of disease, thereby preventing clinicians from considering potentially helpful therapeutic options.

**Methods:**

A retrospective review of the clinical records of 100 young patients meeting POTS criteria was undertaken to determine the frequency of HSD, near-hEDS, and hEDS as well as the frequency of MCAS using consensus-1, conservative consensus-2, and clinical criteria regardless of lab support. Effectiveness of MCAS therapies was assessed in relation to the method of MCAS diagnosis.

**Results:**

From records of 392 patients with orthostatic intolerance syndromes, 100 patients met POTS criteria. The frequency of JHD ranged from 13% using strict criteria of hEDS to 34% using HSD Criteria. The frequency of MCAS ranged from 2% using consensus-1 criteria, to 37% using conservative consensus-2 criteria, to 87% using clinical criteria. Patients diagnosed by clinical criteria with or without the aid of labs responded to therapy similarly to those diagnosed with stricter criteria.

**Conclusion:**

Using overly strict criteria to diagnose conditions which have a high prevalence of co-occurrence misses opportunities for potential therapeutic strategies.

## Introduction

It has been recognized for more than two decades, but only recently become well-established in the past five years, that the conditions of orthostatic intolerance with an underlying contributing etiology of autonomic neuro-immune axis dysfunction (sometimes referred to generally as dysautonomia), what we term here, orthostatic intolerance syndromes (OIS), as well as joint hypermobility disorders (JHD), and mast cell activation syndrome (MCAS) co-segregate in patients who have these disorders (1–5). Residual doubts of this relationship were expressed as recently as 2025 (6, 54). Since then, many reports have firmly established this relationship (7, 8) and some have gone further to propose the various mechanisms by which each induces the development of the other (9–13).

Despite the acceptance of these associations, the prevalence of each entity, relative to the others, has varied. The determination of the prevalence is complicated by the fact that clinical investigators cannot agree on the diagnostic criteria for these conditions and that is true for the OIS (although POTS criteria reigns supreme in the orthostatic intolerance world), JHDs, and MCAS.

MCAS has various established and unestablished clinical criteria that would modify these relative prevalences. For instance, Kohno reported that MCAS with positive laboratory support occurred in 66% of those meeting POTS criteria (14). Had Kohno reported their findings based on “strict” MCAS Consensus 1 diagnostic criteria (15, 16), only 2 patients of 69 would have been labeled as having MCAS. Frustrated by what seems to be arbitrary details and challenging logistics of the Consensus 1 diagnostic criteria, particularly its singular focus on tryptase levels at baseline and during a flare in a very specific manner (15, 16), a sizable group of clinicians caring for patients with MCAS resolved to establish new criteria that included the possibility of considering many additional laboratory measures specific to mast cells. It also included the possibility of establishing the diagnosis with response to MCAS-targeted therapies in the absence of laboratory values. This became the Consensus 2 diagnostic criteria in 2020 (17). Most consensus-2 providers consider having laboratory evidence of MCAS superior to simply relying on clinical response absent laboratory evidence. Thus, there are essentially 2 camps of consensus-2 with the first being thought of as a ‘conservative’ approach. Still, when attempting to satisfy the emphasis on positive laboratory values, this consensus-2 conservative approach can have its own frustrations given that many mast cell mediators are very unstable and fleeting and therefore exceedingly challenging to measure accurately not to mention only accessible in those with the best insurance or financial means. This leaves some clinicians to diagnose and manage patients based on clinical presentation and response to therapy alone or despite laboratory data being within range. This clinical consensus-2 approach might be considered ‘loose criteria’ by comparison.

A similar challenge is found with JHD. Much of this challenge arises from the fact that we currently have a nascent understanding of the biological processes that cause JHD and there is a lack of biomarkers. Until JHD is better understood, attempts at developing and applying criteria will be a somewhat arbitrary act even if by consensus. The 2017 hEDS Criteria were introduced to improve diagnostic specificity but faced criticism for being too stringent, biased against young patients, and failing to adequately capture the multi-systemic involvement of hEDS (18, 19). On the other hand, the definition of HSD as per the same document allowed for alternative assessments of hypermobility beyond the Beighton score (20–22). This broad criteria essentially introduced the possibility for nearly all young patient not meeting the 2017 hEDS criteria to be labelled as HSD even though there is significant overlap with benign joint hypermobility found in the healthy pediatric population. Thus, clinicians and geneticists continued to struggle with their own clinical experience and created modifications as they saw fit much of the time. Some opt to simply diagnose patients with HSD using these rather loose criteria. Others require additional features consistent with hEDS but not the full 2017 criteria effectively creating a diagnosis of “near-hEDS” or “hEDS-in-waiting.” This strategy is supported by the work of Colombi (23).

In 2023, The Pediatric Working Group of The International Consortium on Ehlers-Danlos Syndromes and Related Disorders, addressed what was viewed as a diagnostic bias against children (24). The 2017 Criteria were thus modified to be more appropriate for children. Additionally, the recommendation was made that a diagnosis of hEDS not be made until a patient reaches biological maturity or the age of 18 years. Before that age, the diagnostic process is to be considered fluid with updates as additional signs, symptoms and comorbidities arise. At that age, in place of a diagnosis of hEDS, the use of Pediatric Generalized Joint Hypermobility (PGJH) is now recommended. This is further stratified into 8 categories depending on the presence of core comorbidities and with or without skin or musculoskeletal involvement. Still, this likely temporary solution fails to address young adult patients who still continue to have a bias against diagnosis by the 2017 criteria.

At the time of this publication, the larger body of The International Consortium on Ehlers Danlos and invited international organizations of clinicians, researchers, and patient advocacy groups have been actively working to update the 2017 Criteria entirely to overcome not only shortcoming toward the pediatric and young adult populations but the older adult population as well. According to statements released from the group, this newer iteration will likely take into consideration associated comorbidities such as the “syndromes of ptoses” (see Table 1).

**Table 1:** Conditions included to support the diagnosis of modified HSD (loose criteria) and near-hEDS (conservative criteria) including the “syndromes of ptoses”.

Chiari malformation
Vertebrobasilar insufficiency
Craniocervical instability
Temporomandibular joint dysfunction (minor)
Upper airway resistance syndrome/ obstructive sleep apnea (minor) criteria)
Idiopathic intracranial hypertension or hypotension
Cerebrospinal fluid leak
Scoliosis (minor)
Bowel dysmotility (minor)
Large or small bowel visceroptoses
Median arcuate ligament syndrome
Superior mesenteric syndrome
Nutcracker syndrome
Pelvic congestion syndrome
Nephroptosis
Abdominal hernias (minor)
Pelvic floor, rectal, or uterine prolapse
Tethered cord

Conditions are considered major criteria unless designated minor. Minor criteria would not be used by itself to support a diagnosis.

In the meantime, for purposes of considering prevalence with respect to these other conditions, there remains a desire to identify those of all age ranges with JHD that is beyond that of HSD and approaching a diagnosis of hEDS. For this purpose and while taking into consideration the wide gap between a diagnosis of HSD and full hEDS and the added complexity of how young patients compared to young adults, and to older adult patients are considered, we embrace the concept of Near-hEDS. Our definition of Near-hEDS includes those patients who have not reached biological maturity and meet criteria for Pediatric Joint Hypermobility (24) and those young adult patients who have reached biologic maturity but have not quite met full criteria for hEDS but have at least one related comorbidity that makes it more likely they might meet full hEDS criteria in the future.

Given these challenges, one is left to wonder how to interpret prevalence data for these entities. In 2021, Wang et al. reported that MCAS was present in 31% of those with both POTS and hEDS but 2% in those with hEDS without POTS (8). However, the criteria for their diagnosing MCAS and hEDS was presumably left to various clinicians to make diagnoses by their various practices which might have varied in methods. While it might not be the strictest criteria, presumably, the clinicians were using best clinical judgement and acting best for their patients.

OIS, JHDs, and MCAS are conditions which, left unrecognized and unmanaged, can have devastating consequence for patients’ health, quality of life and longevity. Furthermore, very often the MCAS therapies of histamine blockers and other mast cell stabilizing agents are relatively benign, and so the risk of overprescribing, particularly on a trial basis, is low. On the other hand, overcalling these conditions or labelling another disease process as one of these conditions also can have negative consequences. Determining the most accurate prevalence of clinical disease despite falling short of specific criteria is important to prevent the delay of diagnosis and institution of therapy.

## Methods

### Study participants

We reviewed retrospectively the medical records of patients between the ages of 8 to 25 with orthostatic intolerance as defined by Stewart (25) and Sandroni (26). In order to meet the definition of one more of the syndromes of chronic orthostatic intolerance as defined by Raj et al. (7), at least one additional sign or symptom of dysfunction of the autonomic nervous system (listed immediately below) was required as evaluated by the corresponding author (AJM) in five centers between September 2017 and July 2022 for study inclusion. Patients considered for inclusion were those referred either 1) directly to the cardiologist for evaluation of a three months or longer history of orthostatic intolerance with one or more symptoms of syncope, palpitations, racing heart, dizziness, fatigue, exercise intolerance, shortness of breath, and/or chest discomfort whereby non-autonomic cardiopulmonary causes were ruled out by detailed evaluation or 2) with a longstanding history and documentation of orthostatic intolerance with additional signs of autonomic dysfunction by other expert investigators following their workup. No patients were excluded due to cultural or language barriers. Patients were not provided compensation for their participation.

From the first 392 records reviewed, 100 patients met POTS criteria as most recently defined by Raj et al. (27). See Supplementary Material for the full diagnostic criteria. The clinical and laboratory data of these patients were reviewed and patients were parsed into 4 hypermobility groups based on “No Evidence,” “Loose,” “Conservative” and “Strict” hypermobility criteria, see Table 2.

**Table 2:** Hypermobility groups.

1) No evidence pathologic hypermobility: All patients met criteria for HDS. Those in this category had no additional conditions indicating a pathologic state.
2) Loose: Modified-HSD: meets 2017 criteria for HSD + 1 additional condition from Table 1.
3) Conservative: Near-hEDS: for those beyond biological maturity, meets 2 of 3 criteria per 2017 hEDS criteria +1 additional condition from Table 1. For those prior to biological maturity, meets criteria for PGJH +1 additional condition from Table 1.
4) Strict: hEDS: Meets 2017 hEDS criteria (18)

The 100 patients also were parsed into groups according to “No Evidence,” “Loose,” “Conservative” and “Strict” criteria for MCAS, see Table 3. From these 2 sets of criteria, 3 simple Venn diagrams can be produced to look at prevalence with respect to restrictiveness of criteria for both MCAS and hypermobility. While all clinical diagnoses were performed by the corresponding author, the retrospective parsing of patients into categories according to study criteria was performed by research investigators who had no clinical bias and were blinded to study outcome.

**Table 3:** MCAS groups.

1) No evidence of MCAS
2) Loose: Meets consensus-2 criteria without requirement of positive laboratory data but with clinical criteria including response to therapy
3) Conservative: Meets consensus-22 criteria with emphasis on laboratory confirmation
4) Strict: Meets consensus-1 criteria with modification of tryptase requirement

#### More specific categorizing details are in order beginning with JHD

Strict Criteria: The diagnosis of hEDS is based on the criteria defined by Malfiat et al. (18). This is what we are considering “strict criteria” and, despite the 2023 recommendations of Tofts et al., it is applied equally across all ages for the purposes of this study.

Conservative Criteria: This was a particularly challenging category given the age range of our patients covering both young patients prior to biological maturity and young adult patients still biased against meeting full hEDS criteria. To meet this challenge we present criteria for Near-hEDS. For those patients below the age of biological maturity, Near-hEDS are those who met criteria for PGJH with core comorbidities with or without skin or musculoskeletal involvement (24). For those who have reached biological maturity, Near-hEDS is defined as having met the 2017 hEDS Criteria except meeting only 2 of 3 of the 3 Clinical Criterion (Feature A: Generalized Joint Hypermobility, Feature B: Positive Family History, Feature C: Pain and Joint instability). In place of the missing feature, any one of the major conditions associated with JHD in Table 1 must instead be present while minor conditions were additionally supportive. We anticipate that this added criteria will likely be more in line with the next iteration of criteria presented by the most recognized consensus group.

Loose Criteria: For the diagnosis of HSD, we generally followed the criteria for Generalized (joint) HSD as defined by Castori et al. (20). However, objective scoring of generalized joint hypermobility (GJH) was sometimes challenging. While this was mainly by the Beighton Score, alternative criteria recommended by Malfait et al. was also used including use of the Five Point Questionnaire adapted from Hakim and assessment of alternative joints which have been correlated with the Beighton Score (18, 21, 28). Use of alternative criteria introduced ambiguity in practice that increased the probability of overlap with benign joint hypermobility given the age range being assessed. Thus, those being considered by alternative GJH criteria must also have a condition from Table 1. In addition to the diagnosis of GJH, patients must have one or more musculoskeletal manifestations as described by Castori and fully listed in the Supplemental Section.

#### Additional details for defining the MCAS categories are given here as well

##### Strict criteria

For the “Strict Criteria” for MCAS, we used the published consensus-1 criteria but modified it with respect to the details of the tryptase elevation. For the purposes of this study, a single tryptase value greater than the upper limit of normal for that laboratory was considered positive to satisfy the tryptase requirement to meet our definition of “Strict Criteria”.

##### Conservative criteria

For our “Conservative Criteria,” we followed the rules of the laboratory-based evidence pathway through the consensus-2 criteria (17).

##### Loose criteria

The clinical diagnosis of MCAS included all those who met Consensus 1 and conservative Consensus-2 and also included those with clinical features of MCAS as described by Molderings and Afrin (17, 29–31). Briefly, these clinical features include a history of ease of developing hives, itchiness, skin rashes, dizziness, brain fog, migraines, palpitations, nausea, abdominal pain, shortness of breath, and chest tightness in response to environmental (including heat or cold), food, medication or supplement exposures, chronic dry, itchy burning eyes, easy bruisability, excessive menstrual bleeding, and aseptic cystitis. Physical findings include dermatographism, hives or rashes. When appropriate to support the diagnosis, the Weinstock, Afrin and Molderings Mast Cell Mediator Release Syndrome (MCMRS) Questionnaire and Quick Environmental Exposure and Sensitivity Inventory (QEESI) were completed (32–36). If a clinical diagnosis remained unclear, a positive response to mast cell therapies was used to support presence of disease. Trials of MCAS-targeted therapies included various combinations over at least a one month period of histamine 1 and 2 receptor blockers, mast cell stabilizing agents including leukotriene inhibitors, cromolyn preparations, low dose naltrexone, low dose benzodiazepines, diamine oxidase, and omalizumab, as well as others less commonly used.

Response to therapy was determined by investigators blinded to laboratory data and thus MCAS group assignments. Patients who reported improvements in symptoms and showed quality of life improvements by COMPASS-31 and other validated instruments within a month of initiation of therapy as well as proclivity to ongoing use (report of active use, refill requests) following initiation of MCAS-targeted therapies and reduction in the need for other medications were considered positive responders. Adverse events during these therapies and attributed to such therapies were also recorded.

### Standard protocol approvals, registrations, and patient consents

The study was exempted by an independent review board (Ethical and Independent Review Services) due to its retrospective nature, and informed consent was waived. The corresponding author has full access to all the data in the study and takes responsibility for its integrity and the data analysis.

## Results

### Study participants

One hundred patients met criteria for POTS. Patient characteristics are summarized in Table 4. The great majority of our population was female and Caucasian despite being in regions that are very racially diversified. Of the 95 patients considered for a diagnosis of MCAS, 69 patients were able to obtain Consensus 2 mast cell mediator laboratories. The remainder were unable for various reasons including insurance coverage, priority of other labs sought, and difficulty finding a laboratory willing and able to perform such specialized studies.

**Table 4:** Demographics of subjects with POTS.

Age (years)	Mean: 17.2 +/− 3 Range: 9.6–25
Female (%)	80%
Caucasian (%)	86%
Orthostatic heart rate rise	46 +/− 14

#### Strict criteria

**Figure 1:**
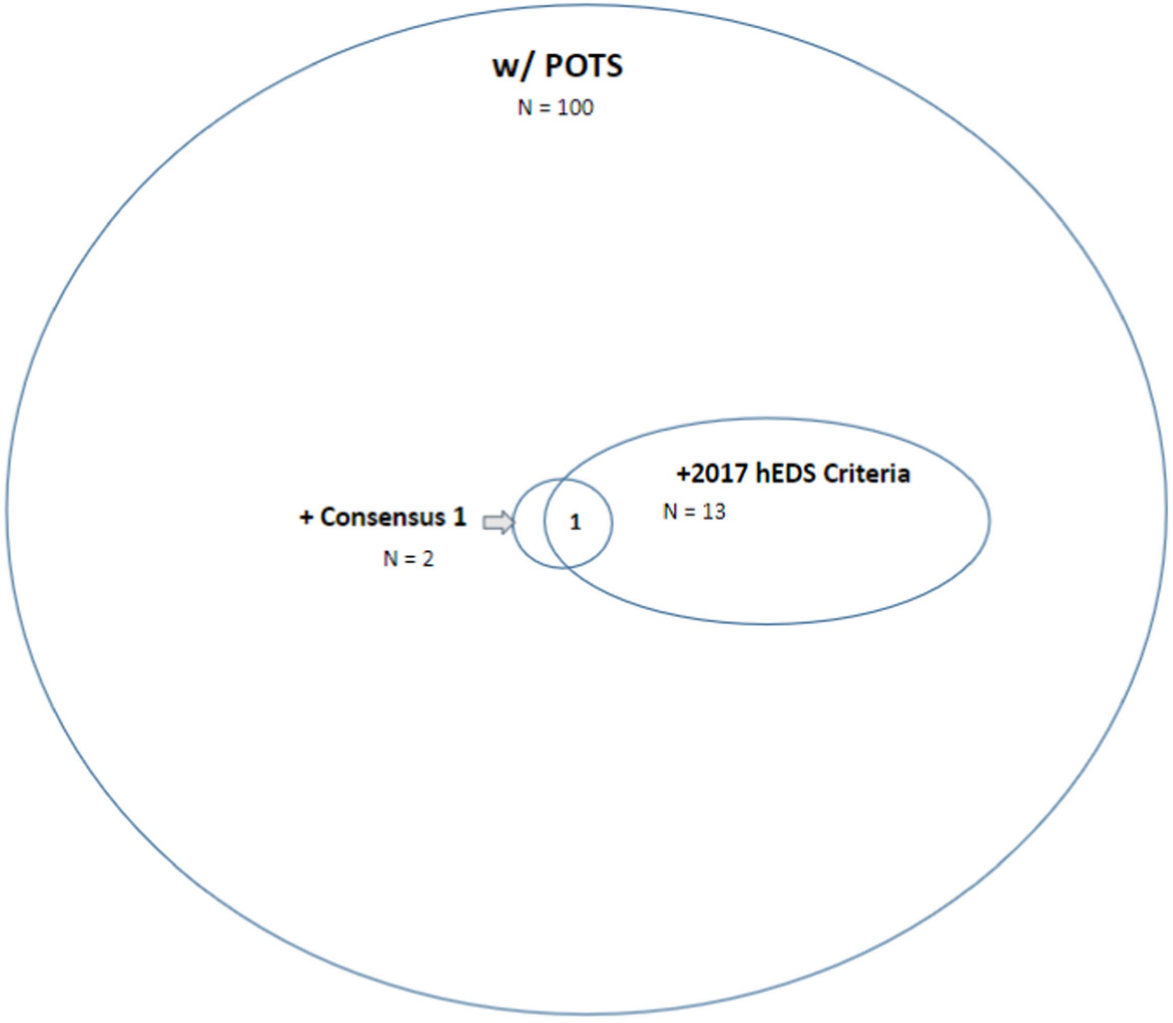
Venn diagram of patients with POTS with or without MCAS based on “strict criteria” of consensus 1 criteria and with or without hEDS based on 2017 hEDS criteria.

Out of 100 patients with POTS, 69 were able to test for tryptase. 2 met strict criteria for MCAS, 13 met 2017 Criteria for hESD and 1 patient had the triad of all 3 (Figure 1). For both patients with elevated tryptase, no other laboratory findings supportive of MCAS were found despite their measure. No patient was able to fulfill the published Consensus 1 requirements of tryptase levels both at baseline and during a flare (37). The single elevated measure of tryptase in 2 of 69 patients (12.1 and 13.5 ng/mL) may be quite in-line with the published frequency of hereditary alpha-tryptasemia (HαT) (38, 39). This was not assessed further in these patients.

#### Conservative criteria

**Figure 2:**
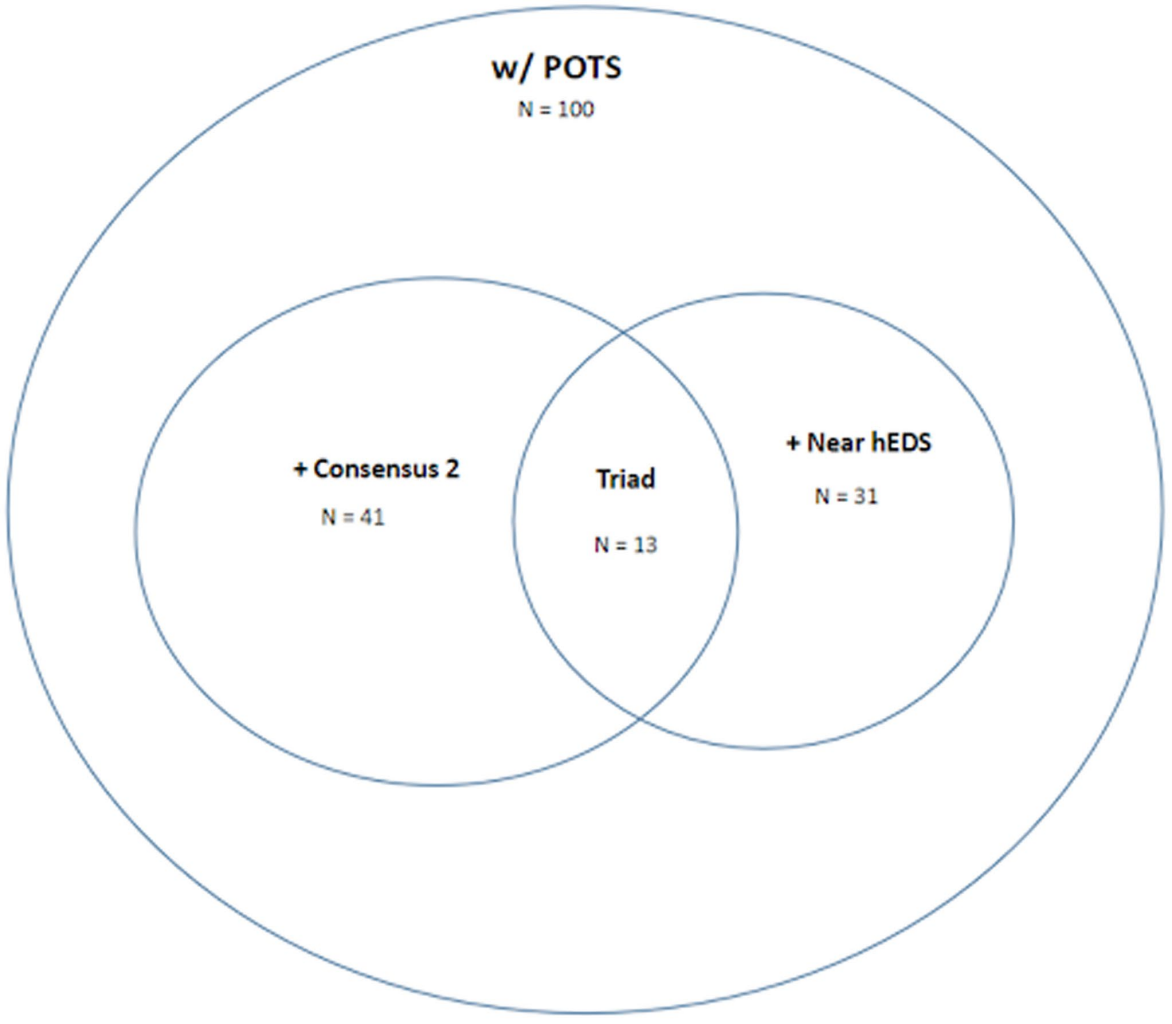
Venn diagram of patients with POTS with or without MCAS based on “conservative criteria” of laboratory-supported consensus 2 criteria and with or without near-hEDS as defined in this study.

Out of 100 patients with POTS, 69 were able to obtain mast cell mediator laboratories. Forty one met criteria for MCAS, 31 met criteria for Near-hESD and 13 patient had the triad of all three. The laboratory values that were positive in the MCAS patients are tallied in Table 5. The most commonly positive lab was plasma histamine followed by chromogranin A. Three patients had three abnormal lab values, 13 patients had two abnormal values, while the remaining 25 had a single abnormal value differentiating them from the Loose Criteria group (see Figure 2).

**Table 5:** Conservative criteria lab values (frequency) – out of 69 patients able to test.

Mediator	# Abnormal
Histamine (plasma)	19
Chromogranin A (serum)	10
CD117 positive staining of duodenal biopsies	8
Prostaglandin D2 (serum)	8
Prostaglandin F2 (serum)	7
N-Methylhistamine (urine)	5
Tryptase (serum)	2
Prostaglandin D2 (urine)	2
2,3 Dinor 11-beta-prostaglandin F2 alpha (urine)	1
Total	62 lab abnormalities in 41 unique patients

Duodenal biopsies stained with CD117 found increased mast cell counts in 8 of 11 patients whose samples were stained. For these patients, the average mast cell count in these biopsies was 48 +/− 25 per high power field (hpf). CD117 staining was the only positive laboratory found in four (10%) of the Consensus 2 patients. Of the three patients staining negative one belonged to the Conservative Criteria group having other positive labs while 2 belonged to the Loose Criteria group.

#### Loose criteria

**Figure 3:**
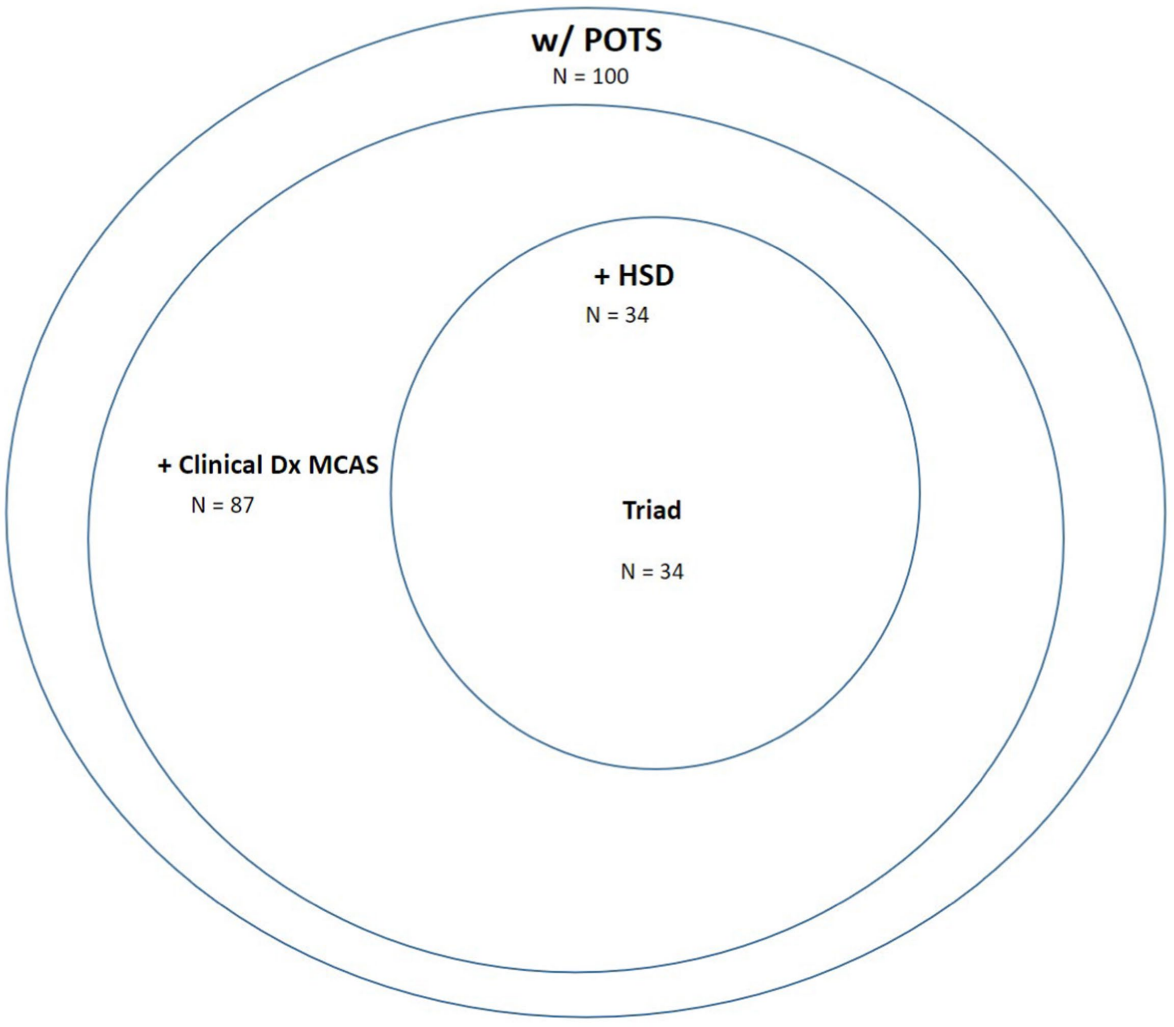
Venn diagram of patients with POTS with or without MCAS based on “loose criteria” of clinically-supported consensus 2 criteria and with or without modified-HSD as defined in this study.

The Figure 3 Venn diagram shows that, out of 100 patients with POTS, 87 met “loose criteria” aka consensus-2 clinical criteria for MCAS, 34 met criteria for JHD as well as the triad of all three. The laboratory values that were positive in the MCAS patients are the same as reported for the Conservative Criteria patients in Table 5. Supporting clinical criteria are tallied in Table 6. Of the 87 patients given a clinical diagnosis of MCAS, all had clinical history, signs and symptoms and physical findings of MCAS as described in Methods. While the MCMRS and QEESI questionnaires were completed in 32 patients with uncertain diagnosis, these were ultimately more useful as a record of specific history, signs and symptom than as a determinant of classification as evident of equally high MCMRS and QEESI scores of those ultimately determined not to have MCAS as those determined to have MCAS. A positive response to MCAS-targeted therapy was an important determinant in 45 of the 46 patients who did not have laboratory markers but determined to have MCAS.

**Table 6:** Criteria to support clinical diagnosis.

Clinical signs and symptoms of MCAS	87
Positive laboratory values c/w MCAS	68 total abnormal, 41 unique patients
Positive MCMRS score > 13	27 of 32
Positive QEESI score > 40	16 of 32
Positive response to therapy	74 (5 NA)

NA = not assessable–either lost to follow-up or patient or investigator unable to determine benefit.

### Response to MCAS therapies

Of the 100 patients with POTS, 5 patients were found to not have any symptoms that merited consideration for the presence of MCAS. Of the 95 patients remaining, symptoms were present that merited the consideration of the presence of MCAS including a trial of MCAS-targeted therapy (see Table 7). No patients trialed on MCAS-targeted therapy experienced any adverse events that were not correctable by simply discontinuing the therapy and sometimes substituting it for another. Following evaluation and a trial of MCAS medications, a total of 87 patients were ultimately diagnosed by any of the criteria used as having MCAS while there were 8 who did not respond and, by weight of the clinical evidence, were determined to not likely have MCAS.

**Table 7:** Response to therapy.

	Response to MCAS-targeted therapy (total assessed = 90)							
	*N*	Age	MCMRS score	QEESI	NA	A	Positive response	No apparent positive response
Total patients trialed MCAS-targeted Rx	95	17.4 +/− 3	17.5 +/− 6	19 +/− 12	5	90	74 (82%)	16
Patients trialed MCAS-targeted Rx absent supporting labs	54	17.4 +/− 3	18.2 +/− 5	21 +/− 10	1	53	40 (75%)	13
Patients with MCAS by clinical diagnosis								
Clinical diagnosis (including positive labs)	**87**	17.2 +/− 3	17.5 +/− 6	17 +/− 14	5	81	74 (91%)	8
Consensus 2 w/lab Diagnosis	41	17.2 +/− 3	17.1 +/− 6	18 +/− 12	4	37	34 (92%)	3
Consensus 2 clinical Dx Absent laboratory support	46	17.0 +/− 3	18.2 +/− 5	21 +/− 10	1	45	40 (89%)	5
Patients determined not to have MCAS by clinical diagnosis								
Considered for MCAS Trialed Rx	8	17.7 +/− 3	19 +/− 3	20 +/− 8	0	8	0	8
No clinical evidence of MCAS throughout	5	17.4 +/− 2	N/A	N/A	N/A	N/A	N/A	N/A

NA = Response not accessible either from lost to follow-up or too soon to determine at last assessment. A = Response to therapy accessible.

Of those diagnosed with MCAS, 81 were able to be assessed for a response to MCAS therapy. The clinical response to therapy was not assessable in 5 patients the records were not clear about a response specifically to MCAS therapy, either because time to assessment was not sufficiently long to be clear or the patient was lost to follow-up (NA). Of those 81, the average time of follow-up since starting therapy was 33 months [23 SD, min 1.6, max 82 months]. At the shorter end of the assessment period, 5 patients were determined to have a positive response with less than 12 weeks follow-up with the shortest follow-up time to positive response being 7 weeks. Of those responding to assessment (A), 74 patients (91%) reported an overall positive response of some type considered related to the MCAS-targeted therapy. Therefore, of 95 patients undergoing MCAS-targeted therapy, 90 assessments were made of response with 74 (82%) patients having a positive response. A subset of this group were the 41 of 69 patients able to obtain laboratories diagnosed using Conservative Criteria. Of these 37 were able to be assessed for response to therapy. 34 patients (92%) were determined to have a positive outcome.

## Discussion

Consistent with other published experiences with OIS, the great majority of our population was female (80%) and Caucasian (86%) despite being in regions that are very racially diversified. Indeed, in a recent report, Boris et al. reported 77.5% female and 93–94% Caucasian in their mainly Philadelphia-based population (40, 41).

This review of clinical data highlights the difficulty in determining prevalence of these often unrecognized and poorly defined conditions particularly in relation to each other. How they are defined greatly determines their frequency in general and with respect to each other. With respect to treatment opportunities, we would argue that broader definitions of both MCAS and HSDs have clinical merit. The rationale for this might be found in how each interacts in the pathophysiology of the others.

There is growing recognition of the role aberrant mast cells play in JHD and in OIS (8, 10, 42, 43). It is becoming clear that these conditions play off and amplify each other. One theory gaining traction is that aberrant mast cells secreting elastases and other proteases break down connective tissue and, in doing so, tenderize ligaments and joints (42, 43). Indeed, this may be the basis for JHD at least in some cases. Our finding that 33 out of 34 patients found to have JHD had clinically apparent MCAS supports this theory. Likewise, aberrant mast cells through various means can cause OIS and, by various pathways, JHD and OIS may lead to MCAS.

Examples of mechanisms of these include the creation of craniocervical instability by way of mast cell-derived elastases tenderization of the ligaments of the cervical vertebrae (44). This is followed by dislocation or subluxation of the first cervical vertebrae (C1) forward causing injury to the pharyngeal plexus thereby causing vagal nerve dysfunction (45). Another example is that aberrant mast cells in the gastrointestinal tract break down the integrity of the epithelium. This may be specifically by mast cell derived elastase-2 breaking down the e-cadherins responsible for cell to cell adherence (46–48). Once this creation of ‘leaky gut’ occurs, inflammation deeper in the tissues ensues, aggravating the afferent (sensory) portion of the vagus nerve leading to disrupted activity of the efferent portion of the vagus nerve with ensuing dysautonomia syndromes of various types (49).

Given the extent of the role that aberrant mast cells play in these intimately-associated conditions, it becomes apparent how prevalent MCAS is when one or both of these conditions are present. It also becomes apparent how important it is to suppress aberrant mast cell activity to (1) arrest further progression of all three entities and their associated conditions, (2) improve the immediate condition and perhaps even (3) allow healing and restoration of what was otherwise thought of as permanently injured tissues.

These theories of involvement of aberrant mast cells in the pathophysiology of these other conditions should alone be a driving force for considering use of ‘loose criteria’ for diagnosis. However, another consideration is the difficulty at obtaining laboratory evidence specific to mast cell activation. Most mast cell mediators tested are extremely fleeting as would be evolutionarily expected given their extreme potency of their effects on other cells, tissues, organs, and systems. Testing for elevated mediators is very challenging for patients and laboratory personnel. Oftentimes patients must go to the laboratory when they are feeling their worst and they must rely on the laboratory technician and the specialized equipment (refrigerated tubes and centrifuges, dry ice packing) to be in top form upon their unscheduled arrival. In fact, laboratory-based diagnosis of MCAS not uncommonly requires repeat visits to get a single positive mediatory value. All the while, the patient is to refrain if possible from some of the very medications that give them relief so as to maximize their “mast cell flare”.

When comparing the Conservative Criteria group to the subset of patients belonging to the Loose Criteria group with or without supporting labs, there is no difference in age, in MCMRS scores, nor in QEESI scores. Responses to therapies were higher in the group diagnosed by Conservative Criteria (92%) compared to Loose Criteria with some obtaining supporting labs (82% of all patients trialed). Both were higher than the subset of Loose Criteria Group where no labs were obtained (75%). However, it is worth noting that 25 of the 41 patients in the Conservative Group (61%) differ from this subset of patients by the presence of only one abnormal laboratory value. Four of these 25 patients (10% of the full Conservative group) were positive only by way of a duodenal biopsy. It is further worth noting that, by this study, it is apparent that 74 of 90 patients with POTS and findings meriting consideration of MCAS (82%), ultimately benefitted and continued use of MCAS-targeted therapy. If the results of the 69 patients subjected to Consensus 2 criteria were extrapolated to that of 95 patients, then one would expect this number to be 54 patients testing positive. Thus, it might be considered that 20 patients (27% of positive responders) missed an opportunity for a positive response.

As such, the real difference in strategies from a clinical standpoint is the consequences of trialing reasonable MCAS-targeted therapies on those who have clinical findings supportive of MCAS but who do not ultimately gain benefit before discontinuing the trial. This must be weighed against the efforts, costs, insurance obstructions, competing lab priorities, and delays of difficult laboratory testing that is often not believed when the results return negative (not to mention often not believed when they return positive) and against the risks of sometimes needing to pursue an endoscopy for duodenal biopsy when no prior sample is available.

Our study supports the idea that if one applies “Loose Criteria” which are not overly-dependent on laboratory evidence to diagnose patients with MCAS, particularly those at high risk for presence of the disease such as those with OIS and HSD, once can anticipate a decent rate of positive response to therapy without much risk of adverse events nor delay in therapy in pursuit of more solid evidence of the presence of MCAS such as laboratory data. Furthermore, such treatment should not interfere with pursuit of, nor mask, alternative diagnoses. Indeed, if patients feel better from MCAS-targeted therapies, they likely would be in a better position to pursue other diagnostic workups and would be better able to tolerate treatments targeting other conditions.

One might argue that use of ‘loose criteria’ over-diagnoses the condition of MCAS. In our study of POTS patients with a high frequency of JHD, the positive response rate to therapy was at least 75% (in those who obtained no supporting labs) and the frequency of considering MCAS was 95%. Therefore, the potential to over-diagnose could be no more than 20% and this is mostly corrected by a lack of response to therapy within the first few months of trialing medication. Those who experience a placebo effect of such therapy might prolong therapy longer but ultimately will declare themselves unresponsive. These patients are also the most likely to not have adverse effects of medication use.

A counter-argument might be that most of these patients with JHD and OIDS have at least some low level of mast cell activation, if not full-fledged MCAS by any criteria. Indeed, this mast cell activation at whatever level is likely aggravating these other conditions, if not the principle driving force behind them. It is worth considering that all patients with these conditions can benefit from at least a trial of mast cell-targeted therapy regardless of their initial presentation. Whether positive responders should be labeled as having MCAS might be a matter for further consideration.

### Limitations

This study was limited specifically to those with OIS that meet POTS criteria. For our study set-up, we believe to adequately assess prevalence of three poorly-defined conditions, it would require at least one of them to be well-defined and limited in scope. For that reason, we confined the study to those meeting criteria for POTS. However, there is a much broader group of patients with OIS who do not demonstrate the phenomenon of POTS but are just as ill (three quarters of our patient population with OIS did not meet formal POTS criteria and this is found in other’s populations as well) (50). These include OIS with and without myalgic encephalomyelitis/chronic fatigue syndrome. These patients likely have similar rates of JHD and MCAS and a separate but otherwise similar study might be worthwhile.

Another limitation of this study is what might seem to be arbitrary categories for JHD. We counter that, until the biological processes, pathophysiology(ies) of this broad range of conditions is better understood and biomarkers are available, all attempts at establishing criteria will be somewhat arbitrary –even if by consensus. Furthermore, we found the use of the criteria for HSD, as published, including with alternative methods of diagnosing GJH resulted in a majority of POTS meeting that criteria. This was not particularly useful given that it was often not clear whether some of these patients had pathological HSD or simply benign joint hypermobility or even normal childhood variability in joint mobility. For this reason, we felt it necessary to add additional criteria when using alternative criteria for GJH to parse out those with likely pathologic HSD. Those interpreting these data simply need to keep this in mind.

We also found that use of the 2017 criteria resulted in very few patients meeting that criteria even though it was clear that these young patients were well on their way to meeting such criteria if given another decade or two. Following the publication of that criteria, providers and geneticists have been using terms such as near-hEDS for these patients or, alternatively, declining to give a diagnosis at all given the failure to meet full criteria, thus prompting the desire to have a Near-hEDS category. This deficit has been recognized by the current international consortium who has signaled that they intend to add to the criteria comorbidities such as those found in Table 1 albeit likely with a weighting system. Furthermore, at a recent roundtable discussion by JHD experts at the annual conference of the EDS Canada Foundation, the addition of Table 1 specifically is being considered as new criteria for their recommendations (51). Thus, what might seem an arbitrary criteria for Near-hEDS, is, in our view, an anticipation of upcoming recommendations.

Six years into our data collection, another level of complexity was added with the publication of the recommendations of the Pediatric Working Group (24) with new criteria and the recommendation of a labelling a new entity (subcategorized into 8 new entities) that is neither HDS nor hEDS. In our view, this is a consensus recommendation on criteria for Near-hEDS in young patients. Considering these criteria for use in this paper was problematic. One major feature added to diagnose Pediatric Generalized Joint Hypermobility (PGJH) were comorbidities that were likely symptoms of OIS. Thus, nearly by definition all of our patients in this study met the criteria of this feature. To use it would be double-dipping of signs and symptoms and thus expanding the number of patients found to have met this category. The other problem was that this category only applied to those patients not having reached biological maturity. The remainder of our patients, young adults, were still biased against meeting full 2017 criteria for hEDS. What we found in our study is that if we were to apply the published criteria for PGJH, the same patients that met this criteria met our criteria for Near-hEDS used for our young adults. This makes sense as they either met the criteria for co-morbidities of PGJH or they met Table 1 criteria given the significant overlap.

One might find fault with somewhat circular reasoning of basing the Loose Criteria on therapeutic response, although not entirely required for the diagnosis, and then reporting a high positive response to therapy. Bias might also be introduced into this study given that the corresponding author of this paper is an author of the Consensus-2 publication (17). Every effort was made to minimize any bias through the use of research investigators that had no clinical bias and were blinded to the categories assigned when determining therapeutic outcome. While a formal prospective study to look at the rate of success and failure of MCAS-targeted therapy trials is worthwhile, we report from this study that none of these “treatment failure” patients had any adverse effects of such a trial, nor, as far as we are aware, were they delayed in the diagnosis of alternative causes of symptoms, refuting some contentions in the literature of harm from over-diagnosis (52, 53).

## Conclusion

This review of clinical data highlights the difficulty in determining prevalence of these often unrecognized conditions particularly in relation to each other. How they are defined greatly determines their frequency and, in turn, utilization of therapies. At least in the population of patients with OIS, broader definitions appear to have little negative consequences.

## Data Availability

The raw data supporting the conclusions of this article will be made available by the authors, without undue reservation.
