# Supplementary material for: Association of postural orthostatic tachycardia syndrome, hypermobility spectrum disorders, and mast cell activation syndrome in young patients; prevalence, overlap and response to therapy depends on the definition
Source: Front Neurol. 2025 Apr 25;16:1513199. doi: 10.3389/fneur.2025.1513199 (PMC12063504; doi:10.3389/fneur.2025.1513199)
Supplement: Supplementary file 1 [file Supplementary_file_1.docx]

**Supplemental Section**

Criteria for diagnosis POTS for purposes of this study are defined by Raj et al., 2022. (Raj et al., 2022)

The following criteria must be met:

1. Sustained heart rate increase of >= 30 beats/min or 40 beats per minute if the patient is 12 to 19 years of age within 10 minutes of upright posture.
2. Absence of significant orthostatic hypotension (magnitude of blood pressure drop >= 20/10 mmHg).
3. Very frequent symptoms of orthostatic intolerance that are worse while upright, with rapid improvement upon returning to a supine position. Symptoms vary between individuals, but often include lightheadedness, palpitations, tremulousness, generalized weakness, blurred vision, and fatigue.
4. Symptom duration >= 3 months.
5. Absence of other conditions that could explain sinus tachycardia including
   1. Acute hypovolemia
   2. Anemia
   3. Orthostatic hypotension
   4. Endocrinopathy
      1. Adrenal insufficiency
      2. Carcinoid tumor
      3. Hyperthyroidism
      4. Pheochromocytoma
   5. Adverse effects of medications
   6. Pain attacks and severe anxiety
   7. Prolonged or sustained bed rest
   8. Recreational drug effect

Criteria for Diagnosis Hypermobility Spectrum Disorder for purposes of this study are as defined by this study is as defined by Castori et al. (Castori et al., 2017) for the diagnosis of Generalized (joint) HSD. This begins with the identification of GJH first by the application of the Beighton. The cut-offs for positive Beighton score is 6 of 9 points for pre-pubertal children and 5 of 9 points for those post-pubertal. In some patients, where the Beighton score was equivocal or historical hypermobility was later replaced by decreased mobility as seen in hEDS, or hypermobility is noted in other joints, alternative scoring was applied as described by Hakim and Grahame and Smits-Engelsman et al. In this case, two or more positive responses to the 5 specific questions [(1) Can you now (or could you ever) place your hands flat on the floor without bending your knees? (2) Can you now (or could you ever) bend your thumb to touch your forearm? (3) As a child, did you amuse your friends by contorting your body into strange shapes or could you do the splits? (4) As a child or teenager, did your shoulder or kneecap dislocate on more than one occasion? (5) Do you consider yourself ‘double-jointed” (Hakim and Grahame, 2003)]. Other joints could substitute for joints of The Beighton Score including the temporomandibular joint, shoulder, hip, foot, wrist, ankle, and other digits (Smits-Engelsman et al., 2011).

Additionally, one or more musculoskeletal manifestations must be present. Evidence of joint micro- or macro-trauma, degenerative joint and bone disease, disturbed proprioception, muscle weakness, and other musculoskeletal traits including: pes planus, valgus deformity of elbows, hind-feet, and halluces, scoliosis, accentuated dorsal kyphosis, and lumbar lordosis, and deformational plagiocephaly (Morlino et al., 2017). When alternative methods of assessing GJH were employed or when the presence of a musculoskeletal manifestation was equivocal, we required an additional condition listed in Table 3 be present.

Castori, M., B. Tinkle, H. Levy, R. Grahame, F. Malfait, and A. Hakim. "A Framework for the Classification of Joint Hypermobility and Related Conditions." *Am J Med Genet C Semin Med Genet* 175, no. 1 (Mar 2017): 148-57. <https://dx.doi.org/10.1002/ajmg.c.31539>.

Hakim, A. J., and R. Grahame. "A Simple Questionnaire to Detect Hypermobility: An Adjunct to the Assessment of Patients with Diffuse Musculoskeletal Pain." *Int J Clin Pract* 57, no. 3 (Apr 2003): 163-6. <https://www.ncbi.nlm.nih.gov/pubmed/12723715>.

Morlino, S., C. Dordoni, I. Sperduti, M. Venturini, C. Celletti, F. Camerota, M. Colombi, and M. Castori. "Refining Patterns of Joint Hypermobility, Habitus, and Orthopedic Traits in Joint Hypermobility Syndrome and Ehlers-Danlos Syndrome, Hypermobility Type." *Am J Med Genet A* 173, no. 4 (Apr 2017): 914-29. <https://dx.doi.org/10.1002/ajmg.a.38106>.

Raj, S. R., A. Fedorowski, and R. S. Sheldon. "Diagnosis and Management of Postural Orthostatic Tachycardia Syndrome." *CMAJ* 194, no. 10 (Mar 14 2022): E378-E85. <https://dx.doi.org/10.1503/cmaj.211373>.

Smits-Engelsman, B., M. Klerks, and A. Kirby. "Beighton Score: A Valid Measure for Generalized Hypermobility in Children." *J Pediatr* 158, no. 1 (Jan 2011): 119-23, 23 e1-4. <https://dx.doi.org/10.1016/j.jpeds.2010.07.021>.
